# Network science applied to forest megaplots: tropical tree species coexist in small-world networks

**DOI:** 10.1038/s41598-020-70052-8

**Published:** 2020-08-06

**Authors:** Julia Sabine Schmid, Franziska Taubert, Thorsten Wiegand, I-Fang Sun, Andreas Huth

**Affiliations:** 1 Department of Ecological Modeling, Helmholtz Centre for Environmental Research - UFZ, Permoserstr. 15, 04318 Leipzig, Germany; 2 German Centre for Integrative Biodiversity Research (iDiv) Halle-Jena-Leipzig, 04103 Leipzig, Germany; 3 Department of Natural Resources and Environmental Studies, National Dong Hwa University, 97401 Hualien, Taiwan; 4 Department of Mathematics/Computer Science, Institute for Environmental Systems Research, University of Osnabrück, 49076 Osnabrück, Germany

**Keywords:** Cancer, Cancer imaging

## Abstract

Network analysis is an important tool to analyze the structure of complex systems such as tropical forests. Here, we infer spatial proximity networks in tropical forests by using network science. First, we focus on tree neighborhoods to derive spatial tree networks from forest inventory data. In a second step, we construct species networks to describe the potential for interactions between species. We find remarkably similar tree and species networks among tropical forests in Panama, Sri Lanka and Taiwan. Across these sites only 32 to 51% of all possible connections between species pairs were realized in the species networks. The species networks show the common small-world property and constant node degree distributions not yet described and explained by network science. Our application of network analysis to forest ecology provides a new approach in biodiversity research to quantify spatial neighborhood structures for better understanding interactions between tree species. Our analyses show that details of tree positions and sizes have no important influence on the detected network structures. This suggests existence of simple principles underlying the complex interactions in tropical forests.

## Introduction

Tropical forests are ecosystems of global relevance. Besides their important role in the global carbon cycle^1,2^, they are known for their high species richness^3^. Several hundreds of tree species, often with similar resource requirements, are able to coexist at a local scale over centuries^4,5^.

To better understand the mechanisms that allow for coexistence of tree species in tropical forests, a closer examination of tree interactions is essential^6^ as the species interaction structure is closely linked to the dynamics and structure of the forest community. Various approaches—ranging from theoretical^7,8^ over statistical^9–11^ and pattern-based^12–14^ to mechanistic modeling^15–18^—have been used to describe interactions between individual trees and species in ecosystems. Trees compete for light, space or nutrients within their local neighborhoods^19–22^ and thus tend to interact primarily with nearby neighbors. Thus, the spatial proximity network of trees contains key information on the potential of trees to interact.

Long-term monitoring plots (e.g., of the CTFS‒ForestGEO network^23–26^) facilitate the in-depth analysis of the interaction structure in tropical forests. Besides tree species and stem diameter, also the position of trees within the forest plot is recorded, which allows to study individual trees in their local neighborhoods. We follow here the long tradition of distance-dependent analyses of species interactions in forests mediated by neighborhood competition for nutrients, space or light^19–22^. A potentially powerful and natural approach to analyze proximity and potential interactions between neighboring trees in such data sets is network analysis that has already been applied in numerous disciplines such as computer science^27^, sociology and psychology^28–30^, neurosciences^31^ and ecology^32–34^. Fuller et al.^35^ conducted network analyses for small plots in a tropical forest to assess the impact of tree size on the species composition of its neighborhood in the understory. Here, we apply network analysis for the first time to CTFS‒ForestGEO mega plots (25–50 ha), using all trees with diameter at breast height (*dbh*) larger than 10 cm for the analysis of the spatial proximity networks of trees and tree species to assess the potential for species interactions.

In network analysis, systems are characterized by nodes (here, trees or species) and edges (which represent connections between nodes). Thus, for a tropical forest we can construct a spatial tree network by analyzing the overlapping of the ‘interaction zone’ of individual trees, given as a multiple of their crown size derived from allometric relationships. Symmetric neighborhood interactions (e.g., competition for space or nutrients) result in undirected networks where an interaction occurs if the interaction zones of two trees overlap. In contrast, suppression of trees due to asymmetric competition (e.g., competition for light) leads to directed networks where an edge links the larger ‘overtopping’ tree with the smaller ‘overtopped’ one. However, our focus here is on the non-spatial species networks that are constructed on top of the marked tree networks (with the mark “species”) by combining trees (nodes) of the same species. With this approach a species pair is connected if the interaction zones of at least one pair (or a larger number of pairs) of trees overlap. For example, while point pattern approaches^12–14^ to quantify species interactions rely on mean neighborhood densities, here we focus on proximity of individuals which is the precondition for interactions to occur. The tree and species networks should capture essential features of the interaction structure in tropical forests, given that competition for space and light are main driving forces of forest structure and dynamics^36–39^.

Of special biological interest is the node degree distribution *P*_s_ (*k*) of the species network that tells us in detail how many connections (*k*) the different species maintain with other species. However, it is difficult to derive a priori biological hypotheses on the shape of *P*_s_ (*k*) because the species network emerges from the marked spatial tree network in possibly complex ways through the interacting effects of the distribution of species abundances, tree sizes, and the small-scale placement of trees. Frequently encountered network structures include scale-free networks that show a power law node degree distribution^40^. Such networks show typically few nodes (i.e., species) with many connections to other species, many nodes with few connections and maintain its structural attributes independently of network size. Another property of numerous real-world networks is the ‘small-world’ attribute, which means that there is always a short connection between two randomly chosen nodes (i.e. species), although most nodes are not connected to each other^41^.

By applying network analysis to trees in tropical forests, fundamental questions can be raised: 1. Which type of network structures emerge in tropical forests? 2. Which attributes of the forest drive the observed network properties (species abundance, species identity, tree size, and spatial location)? To answer these questions, we translated forest inventory data into proximity networks of trees and species from three large tropical forest sites in Panama, Sri Lanka and Taiwan. The results will help us to better understand the factors that govern the interaction structure of tropical forests and thereby its assembly and dynamics.

## Results

**Figure 1:**
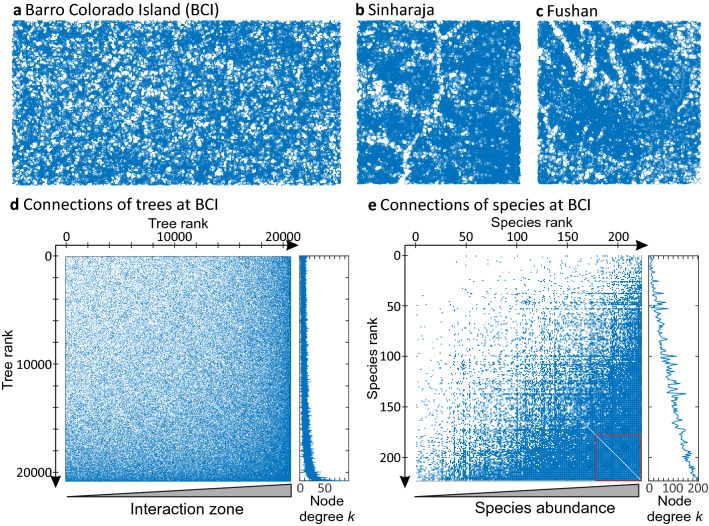
Visualization of the spatial tree networks for the tropical forest plots of (**a**) BCI in Panama, (**b**) Sinharaja in Sri Lanka and (**c**) Fushan in Taiwan. The positions of the visualized nodes correspond to the spatial positions of the trees. Connections in the networks are represented by adjacency matrices: in (**d**) for the tree network and in (**e**) for the species network (50-ha plot). Rows and columns show existing nodes. Nodes are ordered in the tree network by their interaction zone (tree rank shows low values for the tree with the smallest zone to high rank values for the tree with the tallest zone). In the species network nodes are ordered by their abundance (observed number of trees of a species; species rank shows low values for the species with lowest abundance to high values for the species with highest abundance). A blue dot reflects an existing connection (edge) between a pair of trees or species (the specific node in the row and the node in the column). The small panels along the y-axis show the node degrees of (**d**) individual trees and (**e**) species. The adjacency matrices of symmetric connections are symmetric. The adjacency matrix of the tree network reveals 0.5% existing tree connections, while the adjacency matrix of the species network shows that 38% species connections of all possible connections (edges) occur at BCI. In (**e**) the red rectangle highlights that the 50 most abundant species all interact with each other.

For each forest we constructed networks of trees (with individual trees as nodes, Fig. 1a–c) and species (with tree species as nodes, see Methods and Supplementary Fig. S1 for details). In the following we present mostly results of undirected networks (for results of the directed networks see Supplementary Results). Tree networks were about factor 50 to 250 larger than their corresponding species network (Table 1, Fig. 1d–e). The main component (largest connected part) of the tree network at the 50-ha plot of Barro Colorado Island (BCI) in Panama included 20,730 out of 20,735 trees (nodes) which means that almost all trees of the forest were connected (in the following the term ‘network’ refers to the main component). Similarly, all nodes of the species network build one component.

**Table 1:** Network characteristics of tree and species networks at three tropical forest sites.

	Forest site	*N*	< *k* >	*D*	Local connectivity		Global connectivity		Type
					*C*	*C_ER_	*L*	*L_ER_	
Tree network	BCI	10,161	9.6	0.00095	0.631		22.6		
	Sinharaja	17,015	19.2	0.00113	0.635		21.3		
	Fushan	17,647	18.3	0.00104	0.630		22.6		
Species network	BCI	208	65.4	0.316	0.772	0.314	1.69	1.68	SW
	Sinharaja	177	64.7	0.368	0.810	0.367	1.64	1.63	SW
	Fushan	75	37.4	0.506	0.856	0.513	1.50	1.49	SW

To obtain comparable network characteristics among plots we report here results from the left 25-ha subplot of BCI. The characteristics of the other 25-ha subplot and entire 50-ha plot of BCI are similar (see Supplementary Table S1^,^Supplementary Fig. S14). For the results of directed networks see Supplementary Table S10.

*N* number of nodes, < *k* > mean node degree, *D* network density, *C* clustering coefficient, *L* average path length, SW small-world property.

*Clustering coefficient and average path length of random graphs following the Erdős–Rényi (ER) model of the same size (*C*_ER_ and *L*_ER_) for testing the small-world property. See^41^ and Methods for definition of the small-world property.

### Basic characteristics of the tree networks

Tree networks represent spatial (or geometric) networks which are well known in the literature^42–44^. However, in contrast to two-dimensional random geometric networks, where all circles have equal radii and random positions^42^, our tree networks consider different disk sizes (interaction zones) depending on tree crown sizes. A tree individual at the BCI plot was connected on average with approximately < *k* >  = 9.6 other trees (i.e. the average node degree < *k* > ,Table 1). Hence, network density *D* was low (*D* = 0.001 is the observed number of connections divided by the maximal possible number of connections). Almost two thirds of all trees that were connected with a specific tree were also connected with each other (*C* = 0.631 is the clustering coefficient describing the local connectivity, which is close to the value of *C* = 0.587 for random geometric networks^43^). The shortest path between two randomly selected trees passed on average 22.6 other trees (average path length *L*) and no pair of trees required more than 56 other trees to pass (network diameter *d*) (Supplementary Table S1). Most network characteristics (i.e. *D*, *C*, *L* and *d*) were similar between all three tropical sites (Tables 1, Supplementary Table S1). However, tree networks at Sinharaja (Sri Lanka) and Fushan (Taiwan) showed almost the double number of nodes compared to BCI (i.e. they hosted more trees per 25 ha,Table 1). As the mean radius of the interaction zones was similar among all three forests (Supplementary Fig. S2), the higher tree density at Fushan and Sinharaja led also to higher average node degrees < *k* > . Note that the tree size and the species abundance distributions of the three forests were different (Supplementary Fig. S2).

### Node degree distribution of the tree networks

**Figure 2:**
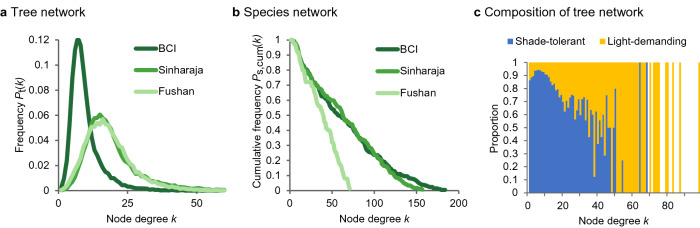
Node degree distributions of the networks and classification of node degrees. (**a**) The node degree distribution of the tree networks *P*_t_ (*k*) with < *k* >  = 9.6, 19.2, 18.3 and *k*_max_ = 83, 136, 88 for BCI (left 25-ha subplot), Sinharaja and Fushan, respectively. (**b**) The node degree distribution of the species networks *P*_s,cum_(*k*) (cumulative distribution) with < *k* >  = 65, 65, 37 for BCI (left 25-ha subplot), Sinharaja and Fushan, respectively. (**c**) Node degrees of the tree network are classified according to shade-tolerant (blue) and light-demanding (yellow) trees (forest site at BCI, entire 50-ha plot).

We found similar patterns of the node degree distribution *P*_t_ (*k*) in the tree networks regardless of the tropical forest site considered. They can be described well by Gamma distributions with a pronounced peak at low node degrees of *k* = 7 (BCI) and *k* = 14 (Sinharaja and Fushan), followed by a decaying tail (Fig. 2a). By assigning either shade-tolerance or light-demanding attributes to each tree individual (node in the tree network; classification based on^45^, we found that the proportion of light-demanding trees increased with increasing node degree (Fig. 2c).

The node degree distributions *P*_t_ (*k*) showed similarities to that known from random geometric networks (with a constant distance threshold between two nodes^42^. However, the distributions of the three forests were somewhat more fat-tailed (Supplementary Figs. S1, S3), probably due to large trees with many connections. Indeed, larger trees (with larger interaction zones) had a substantially higher node degree and interacted mainly with individual trees having high node degrees as well (Fig. 1d, Supplementary Fig. S4).

### Node degree distribution of the species networks

Only 32 to 51% of all species pairs were connected at the three tropical sites (i.e. network density *D*; Table 1) when assuming conservatively that a species connection existed if at least one pair of trees was connected. These figures dropped substantially when requiring up to ten connected pairs for defining a species connection, but stabilized afterwards at roughly 15% for BCI (Supplementary Table S2). The forest with the lowest number of species (Fushan) showed the highest amount of connections (51%), but still half of all possible species connections were missing. Most other characteristics of the species networks were remarkably similar among the three tropical forests (Table 1, Supplementary Table S1).

The node degree distributions *P*_s_ (*k*) of the species networks showed for all three forests the same shape: the *P*_s_ (*k*)’s were evenly distributed, as indicated by an almost linear decline of their respective cumulative frequencies *P*_s,cum_(*k*) (Fig. 2b). This result is surprising because, to the best of our knowledge, constant node degree distributions have not yet been described and explained by network science (see e.g.,^27–34,46^). There is also no a priori reason that the node degree distributions should be structurally similar among forests. The forests show very different species richness, species abundance distributions and ecological characteristics that were crucial for the construction of the networks (see Supplementary Methods, Supplementary Fig. S2).

Figure 1e gives insight into the connection structure of species at BCI based upon their abundance (adjacency matrices). As expected, more abundant species had a substantially higher node degree (see also Supplementary Fig. S5) and connected mainly to species having high node degrees as well. Focusing on the 50 most abundant species reveals that all of them connected with each other (Fig. 1e). In contrast, the adjacency matrix of the directed species network (Supplementary Fig. S4) shows that some species exist (a few of them even with low abundance) which compete asymmetrically with almost all other species (herein referred to as ‘overshadow’).

### Small-world property

Geometric networks such as the here presented tree networks are known a priori not to be small-world^42,47^. In contrast, the species networks of the three forest sites are small-world networks. In agreement with the definition of the small-world property, they have approximately the same average path length *L* and higher clustering coefficients *C* than random networks of the same size^41^ (Table 1, see Methods). This result was robust against more restrictive criteria for occurrence of a connection between two species (e.g., at least eight instead of a single pair of trees must be connected; Supplementary Table S2). Thus, the small-world phenomenon can be observed in tropical forests between species, but not between trees.

Clearly, the species networks are not scale free as they show uniform node degree distributions. Neither a different plot size nor species-specific deviations of interaction zones influenced these conclusions (Supplementary Results). For the analysis of tree networks see Supplementary Results.

### Which factors drive network structures

To determine the degree to which small-scale spatial neighborhood effects influence the observed network properties, we assembled four different types of null communities^48,49^ that randomize certain elements of the observed data. Differences in network properties emerging in the null communities hint to an important role of the randomized elements in structuring the tree community. Our null communities include (1) relocating trees to random positions in the plot (Complete Spatial Randomness—CSR null community), (2) randomly shuffling the species identity among trees (Random Labeling—RL null community), (3) using a fixed tree size for all trees (Equal interaction Diameter—ED null community) and (4) combining CSR and ED to obtain a random geometric network (RGN null community) (see Methods and Supplementary Table S3).

All null communities of the three forests showed species network characteristics that resembled their observed characteristics very closely, including the small-world property (Table 1, Supplementary Table S4), but only with moderate departures due to spatial effects (e.g., intraspecific aggregation and interspecific co-occurrence) present in the observed communities and not in the null communities (Supplementary Table S4, Supplementary Fig. S6). The randomized communities tended to be slightly more connected with larger mean (< *k* >) and maximal (*k*_max_) node degrees, larger clustering coefficients *C* and smaller average path lengths *L*. Interestingly, even the null community based on a random geometric network (RGN) showed only minor deviations from the observed networks, with similarities to the RL communities.

The null communities also approximated the characteristics of the tree networks (Supplementary Table S5), but not as closely as the characteristics of the species networks. Especially the ED and RGN null communities (with constant tree size) showed larger differences to the observed node degree distributions that were more fat-tailed (Supplementary Fig. S6).

## Discussion

In this study, we applied network science to tropical forests by developing a new methodology that translates the size and spatial position of trees into a tree network that measures potential neighborhood interactions between tree individuals by its proximity, and aggregates the tree network into a species network. Our network approach quantifies the complex spatial neighborhood structures occurring in forest communities for better understanding the determinants of interactions among tree individuals and among tree species.

Our first question focused on the types of network structures that develop in tropical forests. We found remarkably similar network structures among forests. One tree was on average connected with ten to twenty other trees. A few tall trees were connected with even more than a hundred other trees. The node degree distributions of all three tree networks followed Gamma distributions, similar to the network of contacts between neighboring linguistic groups^50^. The species networks showed the small-world property with evenly distributed node degree distributions across the three different forest sites, a pattern not described before in network science. The similar structures of the tree and species networks of the analyzed forests (Fig. 2a,b) and the temporal constancy of network characteristics (over up to 30 years of observations, Supplementary Fig. S7) let us hypothesize that the type of networks found here are typical for tropical forests in general.

Only 32 to 51% of all potential pairwise connections between tree species were realized in our most conservative estimates (Supplementary Table S2), which means that not all tree species have the opportunity to interact with each other in the forests simply because they do not meet each other. This result is in agreement with previous studies assessing species interaction strength in tropical forests based on the principle of maximum entropy, stochastic dynamics and spatial point pattern analysis (e.g.,^11, 12,51^).

Interestingly, the Fushan forest with highest tree density and the lowest number of species showed the highest proportion of species pair connections. We can explain this finding on the basis of the results of our null model analyses, which showed that the species abundance distribution drives most characteristics of the species network (Supplementary Table S4). Fushan had the lowest proportion of rare species (Supplementary Fig. S2), species with higher abundance tended to have a higher node degree *k* (Fig. 1e, Supplementary Fig. S5), and therefore a higher proportion of the species pairs were connected at Fushan. Nevertheless, 50 species had less than 100 individuals at Fushan, which explains that still half of the species pairs are not connected.

The lower number of species and the lower proportion of rare species at Fushan have predictable influences on the other network properties of the species networks. First, it causes a higher network density *D* at Fushan, compared to that of BCI and Sinharaja. Additionally, the number *N* of species influences the mean node degree < *k* > (see Eq. (4) in Methods) which is lower at Fushan compared with the two other forests. The maximal node degree *k*_max_ is more strongly driven by the number of species since the most connected species is connected to almost all other species. Still, the local clustering coefficient *C* shows relatively little variation among forests, with a tendency to be higher in forests with lower number of species.

Additionally, species that interact only with few others are as frequent as species that interact with nearly all other species. The observed constant node degree distribution of the species networks is a very particular distribution that differs from the power laws and peaked distributions usually observed in network science^46^. We suspect that this non-standard network type together with the small-world architecture reflect a combination of constraints in forests and a biologically optimal way of species assembly. Potential constraints include tree packing^38^ due to tree architecture together with competition for space and mechanism such as stochastic population dynamics that can generate the typical species abundance distributions of tropical forests with many rare and a few abundant species^52^. The constant node degree distribution is an intriguing pattern, and the consequences of such particular interaction structures for community stability need to be explored. Clearly, the number and type of interactions strongly influences community dynamics and stability, and fewer and weaker interactions can imply more stability^53–56^. Although previous methods required to focus on abundant tree species^11^ or on the understory^35^, we point here to missing interactions that might be highly relevant for understanding species coexistence.

Additional information can be obtained by the distribution of out-degrees (‘shadow indices’) and in-degrees (‘overshadow indices’) (Supplementary Figs. S8, S9, see Methods for details). We found that some species, not necessarily those with the highest abundance, overshadowed many other species. Pioneer species are less likely to be shaded compared to shade-tolerant tree species, but tend to shade other trees (Supplementary Fig. S9). One further feature, especially of the BCI forest, is that the network of light demanding trees fragmented into some 500 isolated components, probably re-colonized canopy gaps^25^, whereas the network of shade-tolerant trees consisted basically of one large component (Supplementary Fig. S10).

Secondly, we investigated which properties of the forest drive the observed network properties. Shuffling species identity among trees, assigning random positions to trees, or setting a constant tree size for all trees resulted in the persistence of the basic characteristics of the species networks, including the small-world property. For example, shuffling the species identities among trees removes species aggregation and the species-specific size distribution and allows each species in principle to have large trees with many connections to other trees. Randomizing tree positions removes effects of species aggregation and small-scale species interactions. Nevertheless, almost all null communities resulted only in a slightly higher network density and average node degree of the species network (Supplementary Table S4). These results suggest that species abundances (together with mean tree size) are the main biological ingredients that determine the overall structure of the species proximity networks (see also Fig. 1e, Supplementary Figs. S5, S11), whereas spatial small-scale patterns of tree placement and tree sizes had only a minor influence on the structure of the species networks (Supplementary Table S4, Supplementary Fig. S6). However, small-scale patterns of tree placement and tree sizes together with the niche overlap between species are important drivers of the performance (e.g., survival, growth) of individual trees (e.g.,^10,48,57^). It is interesting that these effects do not scale up into patterns of the species networks. Thus, it should be possible in principle to derive fundamental aspects of the proximity networks of tropical forests without spatially explicit information of tree positions, only based on species abundance and tree size distributions. This is good news for ecological theory because it tells us that fundamental aspects of forest structure do not depend too much on the idiosyncrasies of the particular local spatial structure.

In this study, we laid an essential foundation for the connection of forest ecology and network science. Our study contributes to the question of determining the interaction structure in ecosystems (e.g.,^11–13^) by taking advantage of powerful methods developed in network science. Tree network analysis thereby allows an in-depth mapping of proximity of trees and tree species. The strong similarities in network structures among different tropical forests is an intriguing pattern that calls for explanation and suggests existence of simple principles structuring fundamental aspects of tropical forests. Perspective applications of our approach further allows to support progress in tropical forest ecology, for example by understanding spread patterns of tree diseases (e.g.,^58^) or the general impact of forest disturbances (e.g., logging, forest fires, or droughts). Linking network science and ecology has a large potential to understand species coexistence and forest ecosystems’ resilience in a globally changing world.

## Methods

### Study sites and tree geometry

We included three field inventories of old-growth tropical forests located on Barro Colorado Island (BCI, Panama), Sinharaja (Sri Lanka) and Fushan (Taiwan) that have plot sizes of 50 ha, 25 ha and 25 ha, respectively^59^. To enable a better comparison of the larger BCI plot with the other sites, we considered in some analyses only the left 25-ha subplot of the BCI plot.

All three plots belong to the CTFS-ForestGEO network^26^ of long-term forest dynamics research sites where all trees are measured in diameter at breast height (*dbh*) are identified to species, mapped, and recensused every five years according to a standardized protocol detailed in Condit^24^. The books Su et al.^60^ and Gunatilleke et al.^61^ provide further specific detail about the inventories at Fushan and Sinharaja, respectively. In the main analysis, we used the data of the 2010 census of BCI^62^, the 2001 census of Sinharaja and the 2013/2014 census of Fushan. To check the generality of our results, we analyzed the node degree distribution of additional censuses of these sites (Supplementary Fig. S7). To derive tree height *h*(*dbh*) in m, we used the allometric relationship (*dbh* in cm):

1 $$h(dbh) = h_{1} \cdot dbh^{{h_{2} }}$$

The parameters *h*_1_ and *h*_2_ in Eq. (1) were derived from independent datasets (^63,64^, see Supplementary Methods for details). We used for our main analyses the same parameters *h*_1_ and *h*_2_ for all individual trees, independent on species identity. For building the tree and species network, we examined only trees with stem diameters ≥ 10 cm and main stems (in case of multiple stems per tree).

### The network analysis

For building the networks, we considered trees as planar disks located at a certain height in a three-dimensional space. The positions of the disks are given by the *x*- and *y*-coordinates of the trees, and the *z*-coordinate is equal to the height of the tree (Supplementary Fig. S1, Eq. (1)). The dimension (or diameter) of a disk $d_{{\text{int}}}$ in m, which represents the interaction zone of a tree, is related to the measured stem diameter *dbh* in cm by the allometric relationship (see Supplementary Figs. S1, S8, Supplementary Table S6 for details):

2 $$d_{{\text{int}}} (dbh) = f \cdot i_{1} \cdot dbh^{{i_{2} }}$$

with parameters *i*_1_ and *i*_2_ derived from tree crown measurements in the field^63,64^ and *f* being a proportionality factor (see Supplementary Methods, Supplementary Fig. S12, Supplementary Table S7 for a sensitivity analysis of the factor *f*). Again, we used the same parameters *f*, *i*_1_ and *i*_2_ for all individual trees.

We tested the sensitivity of the constructed networks to species-specific variations of the parameters in the allometric relationships for tree crowns and tree heights (Eqs. (1) and (2)). Details and results can be found in Supplementary Methods, Supplementary Fig. S13, and Supplementary Tables S8 and S9.

### Constructing tree networks (TN)

For the construction of the tree network (TN), we used all trees which are present within the forest plot as nodes. A network can be either undirected or directed. To construct an undirected tree network, we neglected tree height (height of the disks) and if the interaction zones of two trees (disks) overlap, the corresponding trees are linked through an edge (connection).

In a directed tree network, tree heights are decisive for the direction of the connections (edges). Therefore, we analyzed the forest from a top view and considered shading of each tree (disk) by the interaction zone of larger trees (overtopping disks). Larger trees (higher disks) always have larger interaction zones due to larger tree crowns (granted by the assumed tree allometries, Eqs. (1) and (2)). Hence, if the interaction zones of two trees (disks) overlap, a directed edge links the larger tree with the smaller one (Supplementary Fig. S1). If two trees with the same size (or height) overlap, a random direction of the connecting edge is chosen.

The node degree distribution *P*_t_(*k*) of the tree network quantifies the proportion of nodes with a given node degree *k* (number of connected edges to the node). For a directed tree network, we obtain two different node degrees for each tree (node): the in-degree and the out-degree. As a result, two separate node degree distributions can be calculated. The out-degree of one node reflects the number of trees which are overlapped (or shaded) by the respective tree. The in-degree of one node reflects the number of trees who are overlapping (or shading) the respective tree. The in- and out-degrees can be interpreted as competition indices. We refer to the out-degree as ‘shadow index’ and to the in-degree as ‘overshadow index’ (Supplementary Fig. S8).

The constructed tree networks are geometric networks as their connections arise from geometric rules. However, as we assume different interaction radii (depending on the tree size), the analyzed tree networks differ from classical geometric networks^43^.

### Constructing species networks (SN)

In the tree species network nodes represent all tree species that occur at the tropical forest plot. The species network is obtained from the marked tree network (where the mark represents a species identity) by condensing all nodes of trees which belong to the same species into one species node. That means, at least one overlap between two trees of different species identity results in an edge (connection) in the species network. Again, undirected and directed species networks can be built (Supplementary Fig. S1). If some tree of species A overlaps with trees of species B and vice versa, the emerging species network includes two directed connection (edges) between species A and B (one edge from A to B and the other edge from B to A). Note that the species networks do not belong to spatial networks as their construction does not depend on space anymore. More precisely, species networks (SN) emerge from marked spatial tree networks (TN) by aggregation methods using the additional information of the marks.

### Network characteristics

We used several measures to describe the properties of the analyzed networks. The size of the network is in general expressed by the number of nodes *N* and the number of edges *E*. The density *D* of the network is built upon those values by dividing the number of existing edges by the maximum possible number of edges in the network:

3 $$D = \frac{2}{N(N - 1)}E$$

whereby factor 2 arises for undirected networks, but is dropped for directed networks. The network density is the species network is of special interest because it gives the proportion of species that are connected.

The average node degree $\langle k\rangle$ can be computed by:

4 $$\langle k\rangle = \tfrac{1}{N}\sum\nolimits_{i = 1}^{N} {k_{i} } = \frac{2E}{N}$$

where *k*_*i*_ is the number of edges of node *i* and factor 2 is dropped again for directed networks. The average node degree is the average number of trees a tree is connected with (tree network), or the average number of other species a species is connected with (species network).

The average path length *L* is a global property of the network and indicates the mean of the shortest path lengths *d*_*ij*_ between all pairs *i* − *j* of nodes of the network^65^:

5 $$L = \frac{2}{N(N - 1)}\sum\limits_{i \ge j} {d_{ij} }$$

whereby factor 2 drops again for directed networks. The longest shortest path length *d* = *max*(*d*_*ij*_) is defined as the diameter *d* of the network^66^. The average path length *L* is used to test for the small-world property of a network.

The clustering coefficient *C* is a local property of the network and computed according to Watts and Strogatz^41^ as the average of the local clustering coefficients *C*_*i*_ of all nodes *i* as:

6 $$C = \tfrac{1}{N}\sum\limits_{i = 1}^{N} {C_{i} }$$

where *C*_*i*_ is estimated in analogy to the density *D* of the network as $C_{i} = (2e_{i} )/(k_{i} (k_{i} - 1))$ with *k*_*i*_ being the number of neighbors of node *i*, *e*_*i*_ the number of existing edges between the neighbors of node *i*, and *k*_*i*_ (*k*_*i*_ − 1) being the maximum possible edges between them. Factor 2 drops again for directed networks. For nodes with a node degree of *k*_*i*_ = 1, we defined their local clustering coefficient as *C*_*i*_ = 0. The clustering coefficient is of special interest for local topology of the tree network, because it gives information on the degree of connections among trees that are connected to a specific tree. For the influence of plot size on network characteristics see Supplementary Results.

### Testing for the small-world property of the species networks

For testing the small-world property^28,41,67^, we constructed random graphs that had the same number of nodes and edges as the observed species networks^68^^,^ and compared the clustering coefficient *C* and average path length *L* of the random graphs with those observed. The random graphs were constructed with the Erdős–Rényi (ER) model^68^. A random graph has a node degree distribution that follows a Poisson distribution with expected value being the observed average node degree < *k* > . Furthermore, it generally shows a small average path length *L* and a low clustering coefficient *C* =  < *k* > /*N*.

Small-word networks are networks with properties ranging between regular networks and random graphs^41^. They follow two independent structural features, namely their clustering coefficients are higher than that of a corresponding random graph, while their average path lengths are similar.

In addition to the small-world property, networks can also be ‘scale-free’ if they show a power law node degree distribution. Here, we tested only tree networks for the scale-free property (see Supplementary Results for details) as the constant node degree distribution of the species networks clearly rejects this property.

### Construction and analysis of null communities

We constructed four types of null communities by randomizing one or several of the following elements of the census data: (1) tree positions, (2) species identities, (3) tree size distribution (species-specific), but we maintained the observed species abundance distribution (see Supplementary Table S3 for details).

First, in the Complete Spatial Randomness (CSR) null community^48^^,^ trees were assigned new positions within the forest plot (randomly and evenly distributed). This null model removed the observed species aggregation and co-occurrence patterns, while maintaining the observed identities and the species abundance and species-specific size distributions.

Second, in the Random Labeling (RL) null community, only the existing species identities were randomly redistributed among the trees while keeping the spatial positions of trees, the species abundances and the tree size distribution within the plot. This null model removed the observed species aggregation and co-occurrence patterns as well as the species-specific size distributions, while maintaining the observed species abundances and the overall size distribution.

Third, in the Equal interaction Diameter (ED) null community the observed variable tree sizes were replaced by the constant mean tree size. This null model removed only the observed species-specific size distributions, while maintaining all other properties of the data.

Finally, the Random Geometric Network (RGN) null community combines the CSR and ED null communities by replacing tree sizes by a mean value and by randomizing tree locations.

## Supplementary information

Supplementary Information 1.

## Data Availability

The forest censuses are available at 10.15146/5xcp-0d46 (BCI) and at https://forestgeo.si.edu/ upon request.

## Code availability

Code of the network analysis is available from the corresponding author upon request.
