# Supplementary material for: Network science applied to forest megaplots: tropical tree species coexist in small-world networks
Source: Sci Rep. 2020 Aug 6;10:13198. doi: 10.1038/s41598-020-70052-8 (PMC7413514; doi:10.1038/s41598-020-70052-8)
Supplement: Supplementary file 1 — Supplementary Information 1. [file 41598_2020_70052_MOESM1_ESM.pdf]

Supplementary Information for

Network science applied to forest megaplots: Tropical tree species coexist in small-world networks

Julia Sabine Schmid
Franziska Taubert
Thorsten Wiegand
I-Fang Sun
Andreas Huth

Julia Sabine Schmid
Email: julia.schmid@ufz.de

This PDF file includes:

Supplementary text
Supplementary Figs. S1 to S14
Supplementary Tables S1 to S10
References for Supplementary text

Supplementary Information Text

Supplementary Methods

Geographic conditions of the study sites. The investigated study sites are located in Panama (Barro Colorado Island), Sri Lanka (Sinharaja) and Taiwan (Fushan). All forests are old-growth forests[1,2].

BCI has an annual rainfall of 2551 mm with a severe dry season from December to April or May[2]. The average diurnal temperature maximum is 31.1°C, the minimum 23.2°C. The plot is mostly located on a 140 m above sea level plateau including gentle slopes on the fringes. Unusually severe droughts associated with El Niño events (such as those in 1983 and 1998) are the most important natural disturbance that can provoke high tree mortalities and subsequent canopy openings[3].

Sinharaja is with 5016 mm/year rainier than BCI, due to the missing distinct dry season. With 24.7°C as the average diurnal temperature maximum and 20.4°C as the minimum it is colder and has less temperature fluctuations. The 25 ha plot lies between 424 and 575 m above sea level and encompasses a central valley bounded by two slopes.

In Fushan, the annual rainfall is with 4271 mm/year between BCI and Sinharaja[4]. Due to monsoons and typhoons it is cool and rainy in winter and warm and humid in summer. This results in an average temperature of 18.2°C. The plot has a hill in the western part and a small creek traversing the eastern and southern parts. The woody plant community at the Fushan plot is subject to frequent natural disturbances primarily driven by typhoon induced flooding, landslides, soil-erosion, and wind-induced branch damage[1].

Sensitivity to allometric relationships of the networks. For each tree individual, we chose equal allometric relationships for calculating tree crown diameter and tree height, regardless of species identities. To assess the influence of possible variations among different species, we conducted analyses in which we put noise on (i) tree crown diameters (interaction zones) and on (ii) tree heights (see Methods, equation (1) and (2)). For each species, we therefore multiplied uniformly distributed factors ε_1, ε_2 within a certain range (e.g., between 0.8 and 1.2 for 20 % noise extent) to the parameters of the allometric relationships (for (i): i_1 and i_2, for (ii): h_1 and h_2, see Supplementary Table S6):

$$d_{int}^{\varepsilon}(dbh) = f \cdot (i_1 \cdot \varepsilon_1) \cdot dbh^{(i_2 \cdot \varepsilon_2)} \qquad \text{(S1)}$$

$$h^{\varepsilon}(dbh) = (h_1 \cdot \varepsilon_1) \cdot dbh^{(h_2 \cdot \varepsilon_2)} \qquad \text{(S2)}$$

As the mean interaction diameter $\overline{d_{int}^{\varepsilon}}$ differed with increasing noise, we corrected the calculated values by a linear shift $(-(\overline{d_{int}^{\varepsilon}} - \overline{d_{int}}))$ to obtain in total a mean interaction diameter equal to that of the network without noise ($\overline{d_{int}}$). Note that analysis (ii) affected only the directed networks, as tree heights are not relevant for the undirected networks of the main manuscript.

Both analyses revealed that deviations of tree crown diameters and tree heights from the initial allometric relationships have only minor impact on the resulting tree networks and species networks (Supplementary Tables S8 and S9, Supplementary Fig. S13).

Sensitivity analysis of the interaction zone. The interaction zone (diameter of the tree disk) is assumed to be proportional to the tree crown diameter which is related to the stem diameter of a tree (e.g.,[5], see Methods for details). The proportionality factor f is derived as 1.5, for which all trees (nodes) in the network are connected (e.g., at BCI). Due to the changing insolation angle of the sun during the day and the lateral root expansion an interaction zone larger than 1 (i.e. identical to the tree crown projection area) seems to be reasonable[6]. A factor of 1 would result in a tree network with more than 700 components that means a forest network with many isolated tree clusters independent from each other. Supplementary Figure S12 shows that the shape of the node degree distribution $P_t(k)$ of the tree network and the cumulative distribution $P_{s,cum}(k)$ of the species network are not influenced by the selection of different proportionality factors. In addition, there are only minor changes in the relation between the clustering coefficient C and the average path length L (Supplementary Table S7).

Consequently, the small-world behavior of the species interaction network does not depend on the size of the chosen interaction zone.

Testing the scale-free property. Scale-free networks are characterized by a node degree distribution which follows a power law. To test for this behavior, we fit a power law with exponential cut-off to the logarithmic binned frequencies of node degrees and compared it to a power-law distribution. For the truncated power-law fit we conduct the method proposed by Barabási et al.[7] to find the fitting parameters $k_{\min}$, k_{cut} and γ:

$$p(k) = \frac{(1/k_{cut})^{1-\lambda}}{\Gamma(1-\lambda, k_{min}/k_{cut})} k^{-\gamma} e^{-k/k_{cut}}$$

This includes the combination of a maximal log-likelihood function to estimate γ with fixed $k_{\min}$ and k_{cut} and the identification of $k_{\min}$ and k_{cut} for which the Kolmogorov-Smirnov statistic is minimal. We set a minimum of five binned data points as a condition for the fitting range and compute the root-mean-square error (RSME). To compare the fit with a power law we use the following fitting function with the same $k_{\min}$ value

$$p(k) = \frac{\gamma - 1}{k_{min}} (k/k_{min})^{-\gamma}$$

and analyze the likelihood ratio and Vuong test..

Software used for the analyses. Different software was used for this study. The construction of all networks and calculation of network measures was done in C++ (Embarcadero RAD Studio XE5). With Matlab we created the network visualizations and adjacency matrices (Fig. 1 and Supplementary Figs. S4, S10). The truncated power-law fit and analysis was done with the Matlab packages of Virkar et al.[8]. For plotting the results we used Matlab, R and Microsoft Excel.

Supplementary Results

Results of the directed networks. By construction, the average node degree $<k>$ and network density D of the directed tree networks correspond to half of the values in the undirected case. Concerning the in-degrees ('overshadow indices') we obtain a clustering coefficient of $C \approx 0.35$ and for the out-degrees ('shadow indices') a coefficient of $C \approx 0.16$, which was similar for all forest sites. The outgoing node degree distributions decrease monotonically because there are many small trees that overshadow only few other trees while a few large trees overshadow many small trees (Supplementary Fig. S9a). Consequently, the proportion of shade-tolerant species tend to decline at the BCI forest with increasing out-degree (Supplementary Fig. S9c). In contrast, the incoming node degree distributions rather follow Poisson distributions (Supplementary Fig. S9b).

Note that a maximum value of 0.5 for the clustering coefficient C results from the fact that the directed tree network is acyclic. The difference between the values for the out-degrees and in-degrees can be explained by the typically decaying tree size distribution of undisturbed forests[9,10]. The 'deeper' we look into the forest from above, the more smaller trees and thus, with smaller interaction zones occur. By this, more nodes with an out-degree of $k_{\text{out}} = 1$ or 0 and with a local clustering coefficient $C_{\text{i}} = 0$ are detected (concerning the 'shadow index') which results in a lower global clustering coefficient C.

Considering the species networks, there is no functional relation between the average node degrees of the undirected and the directed networks. The directed species network shows an average node degree of $<k> = 57.55$ at BCI (50 ha), at Sinharaja $<k> = 49.21$ and at Fushan $<k> = 31.27$. For the directed species networks, we obtain a clustering coefficient lying between $C = 0.66$ and $C = 0.74$ for the out-degrees ('shadow index') and between $C = 0.72$ and $C = 0.86$ with regard to the in-degrees ('overshadow index').

Node degree distribution of the tree networks. As expected for node degree distributions that resemble Gamma (or log-normal) distributions, the analyzed tree networks can be considered as thin tailed but not scale-free[7]. An additional analysis showed that a power law with exponential cut-off

approximates the degree distribution better than a power-law distribution (Supplementary Fig. S3), similar to observations in other geometric networks (e.g.,[11,12]).

Over 30 years, the tree data inventory of BCI shows on average a tree mortality of 11.6 % and around 11.4 % tree recruits in every five years (standard deviations s = 0.009 and s = 0.015, respectively). Nevertheless, there is no significant change in the node degree distributions (Supplementary Fig. S7).

Influence of plot size on network characteristics. When plot size was changed (from 50 ha to 25 ha), most network characteristics remained unchanged in the example of BCI.

Some global network properties of the tree network scale in a predictable way with plot size: the number N of nodes (i.e., trees) is proportional to plot size and also the number E of edges since connections between nodes are local. As a consequence, network density D scales proportionally to 1/plot size (see equation (3) in Methods). However, local neighborhood properties such as the mean node degree $<k>$, maximal node degree k_{max}, node degree distribution and clustering coefficient C are independent from plot size (Supplementary Tables S1 and S2, Supplementary Fig. S14a), although the probability is higher to find a node with a higher degree in plots of larger size. The average path length L and the diameter d of a network scales approximately with the increase of the maximal possible distance among points, as shown in Supplementary Table S1.

The scaling with plot size of characteristics of the species network that is constructed on top of the tree network is difficult to predict, except for the number of nodes N that scales with the species-area relationship. A doubling of plot size caused only a slight increase in network size (the number of nodes N and edges E, Supplementary Tables S1 and S2) and in node degrees ($<k>$, k_{max} and node degree distribution). However, the shape of the node degree distribution remained constant (Supplementary Fig. S14b). More connections between the species resulted in a slightly lower average path length L and a slightly higher clustering coefficient C (Supplementary Table S1). The characteristics that change with plot size do not affect our general conclusions about the small-world and scale-free property in the networks of tree individuals and of tree species.

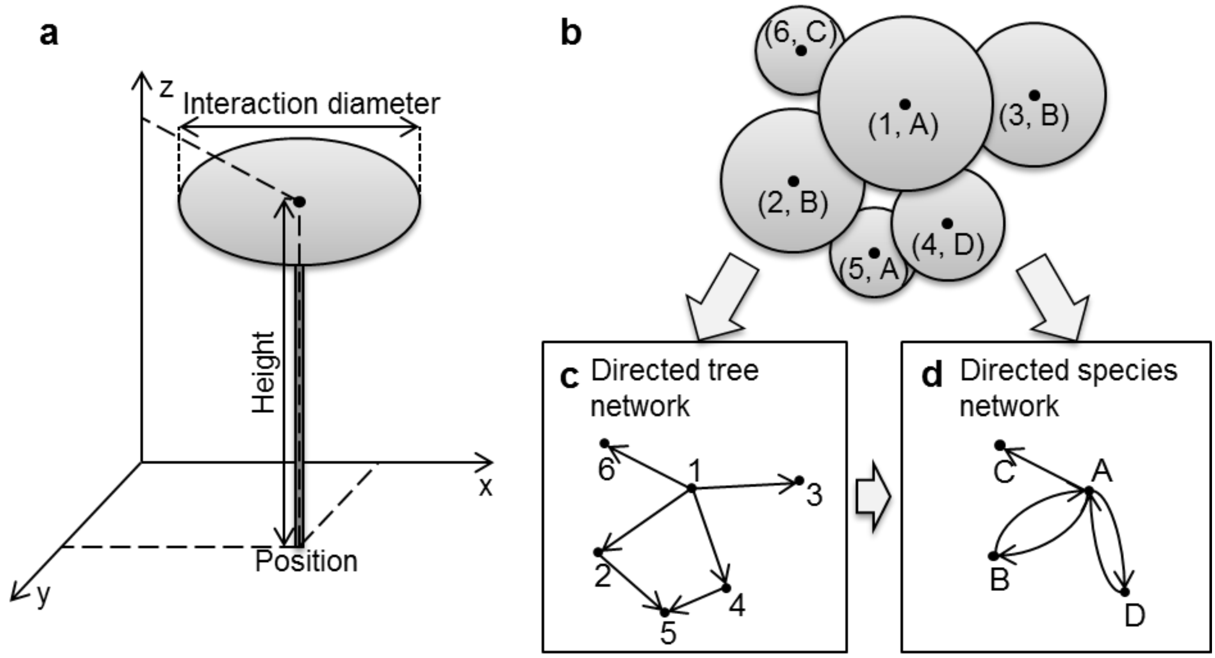

Supplementary Fig. S1 Construction of proximity networks for forests. **a** Visualization of the approach used for the network analysis. Relevant parameters like the tree position, height and interaction diameter of the tree are recorded in forest inventories or derived from measured stem diameters and allometries (see Methods for details). **b** Construction of the directed tree and species networks. Disks show interaction zones of trees from a top view perspective. Numbers in the disks identify single trees and letters their tree species. **c** In the directed tree network, two trees (numbered nodes) are linked from the higher to the lower tree if their interaction zones (disks) overlap. **d** The species network arises by aggregating tree nodes in the directed tree network **c** which belong to the same species (letters A, B, C, D). The edges of undirected networks have no directions.

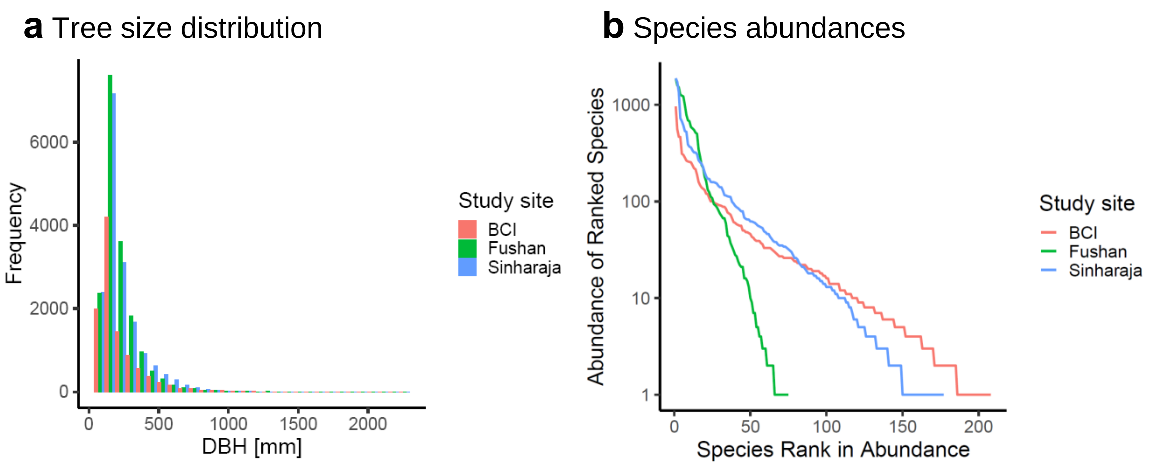

Supplementary Fig. S2 Tree size distribution (diameter at breast height, DBH) and species abundances at all three study sites (BCI 25 ha, left side). Mean tree sizes are DBH = 223 mm (BCI, left side), DBH = 226 mm (Sinharaja) and DBH = 215 mm (Fushan). Mean species abundances are 49 (BCI, left side), 96 (Sinharaja) and 235 (Fushan).

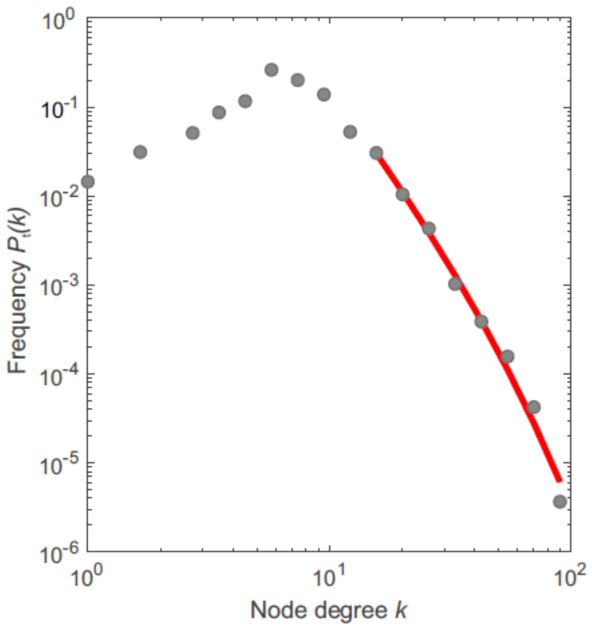

Supplementary Fig. S3 Power-law fit with exponential cut-off (k_{min} = 16, k_{cut} = 48 and γ = 3.408, *RMSE* = 3.08e-04) to the logarithmically binned frequencies of node degrees $P_t(k)$ in the tree network at BCI (50 ha). The truncated power law with exponential cut-off fits the node degrees significantly better than a power-law with same starting value k_{min} (likelihood ratio = -4.52, Vuong test, p = 0.0335, see Supplementary Methods for details). For graphical purposes only, frequencies are normalized (with regard to network size and bin width of node degrees).

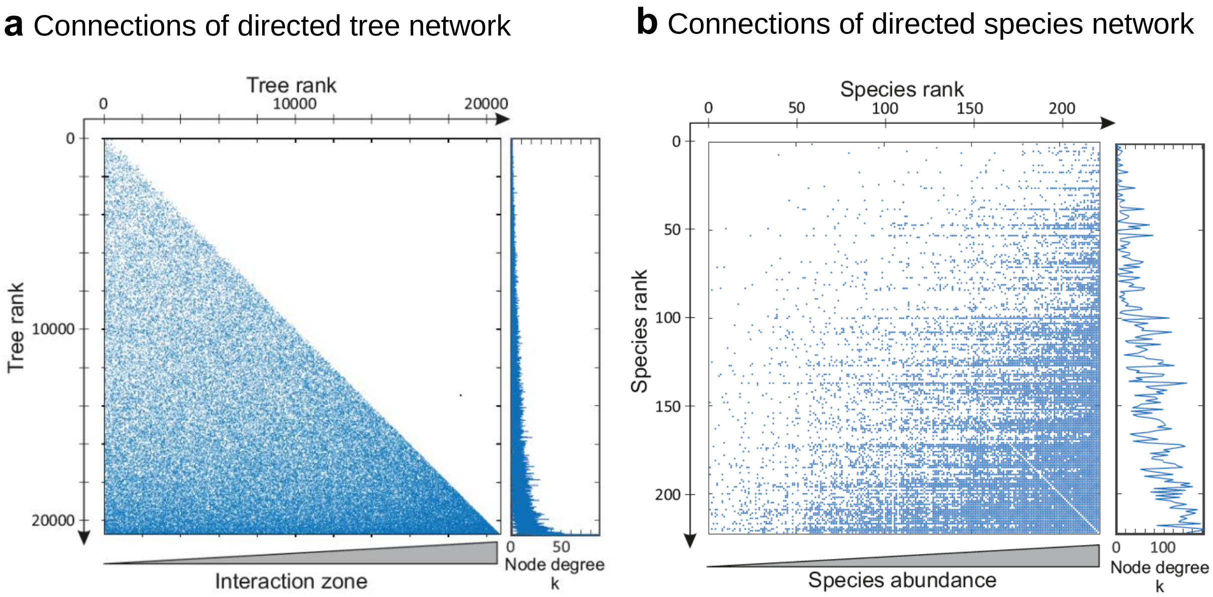

Supplementary Fig. S4 Adjacency matrices of the directed tree and species network for BCI (50 ha). The rows and columns stand for the existing trees or species (nodes). Each blue dot represents a directed connection between a pair of trees or species. Consequently, the number of dots in one row represents the out-degree (‘shadow index’) of the concerning node. Nodes in the tree network in **a** are ordered by tree sizes starting from the smallest tree (low tree rank). Nodes in the species network in **b** are ordered by their species abundance starting from the species with lowest number of trees (low species rank). The small panels along the y-axis show the node degrees of **a** individual trees and **b** species.

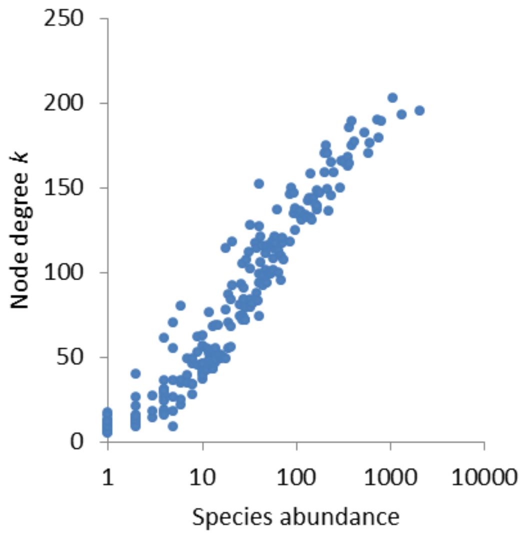

Supplementary Fig. S5 Relation of species abundance to existing node degrees in the species network at BCI (50 ha, year 2010).

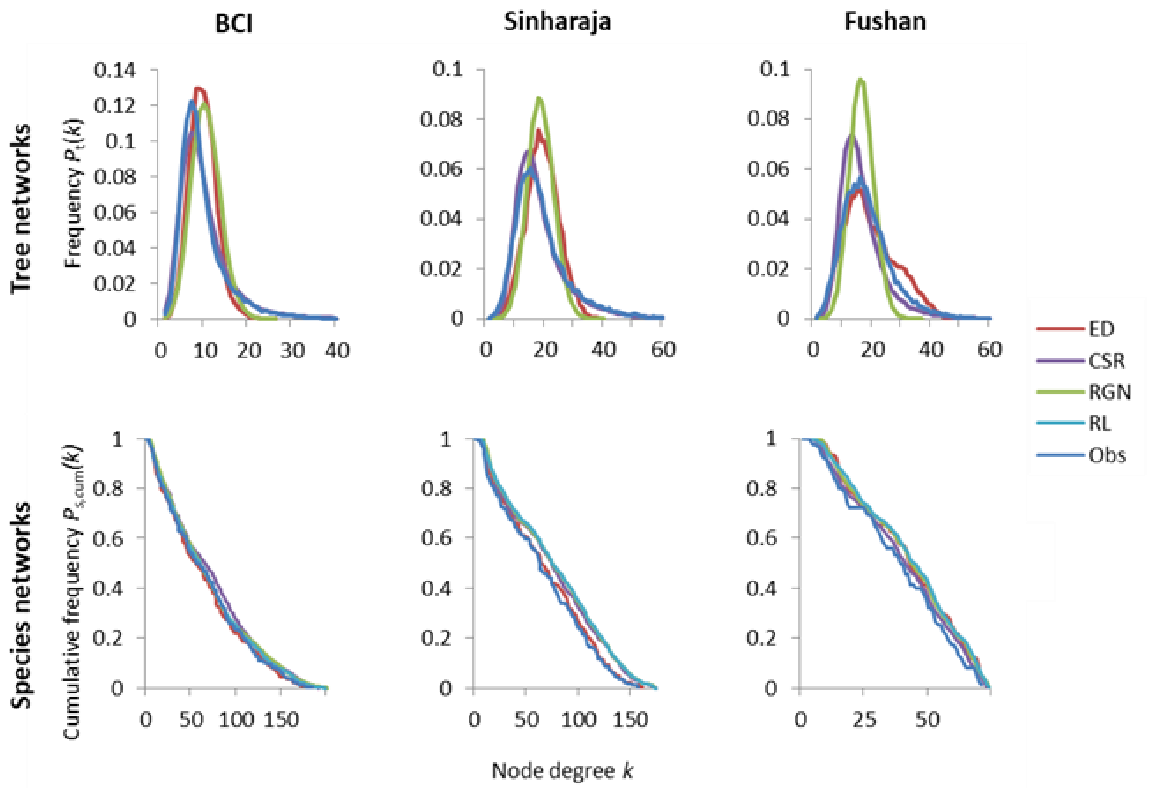

Supplementary Fig. S6 Node degree distributions of the tree networks $P_t(k)$ and the species networks $P_{s,cum}(k)$ (cumulative distributions) for BCI (25 ha, left side), Sinharaja and Fushan (Obs) and their related null communities (ED – Equal Diameter, CSR – Complete Special Randomness, RGN – Random Geometric Network, RL – Random Labeling), respectively. The curves of the null communities are averages of 19 simulations. In the tree networks node degrees are cut at k = 40 or k = 60.

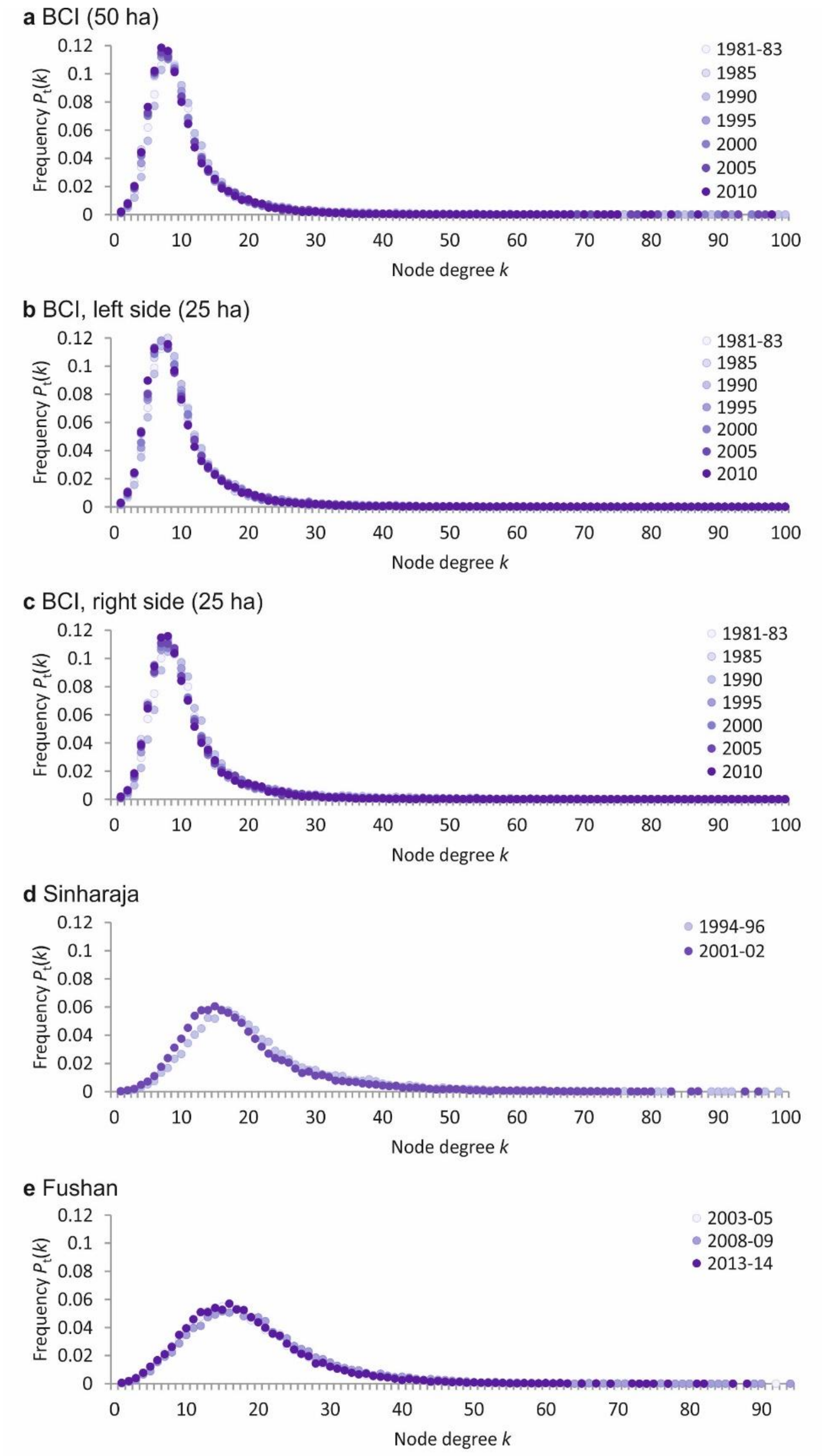

Supplementary Fig. S7 Temporal variations in node degree distributions $P_t(k)$ of the tree networks at **a** BCI (50 ha), **b** BCI, left side (25 ha), **c** BCI, right side (25 ha) censused in years 1981 to 1983, and every five years from 1985 to 2010, at **d** Sinharaja censused in years 1994 to 1996 and 2001 to 2002 and at **f** Fushan censused in years 2003 to 2005, 2008 to 2009 and 2013 to 2014. For graphical purposes node degrees have been cut at k = 100.

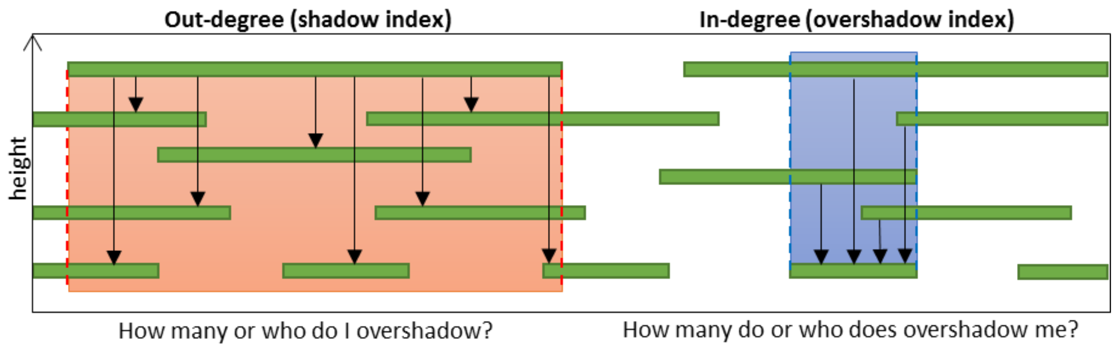

Supplementary Fig. S8 Out-degree (‘shadow index’) and in-degree (‘overshadow index’) derived for the directed tree network. Green horizontal bars represent single trees in the forest (side view). Orange and blue areas visualize the interaction zones of the tallest tree and one of the smallest trees. Arrows show the directed connections for both focal trees (always going from the top to the bottom). The out-degree of the focal tree on the left side is eight (and its in-degree is zero), while the in-degree of the tree on the right side is four (and its out-degree is zero).

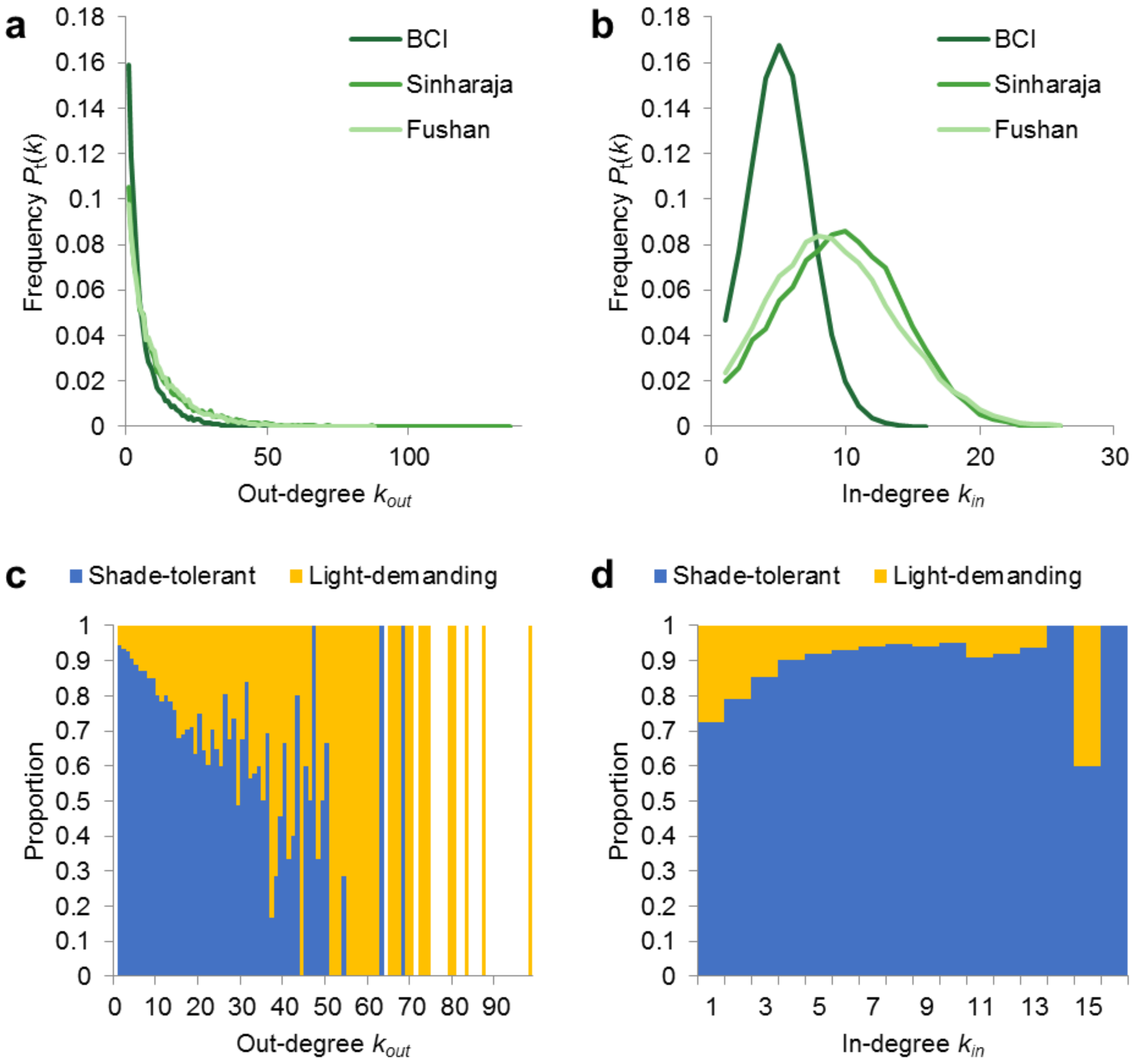

Supplementary Fig. S9 Node degree distributions $P_t(k)$of the directed tree networks **a** reflecting the out-degrees k_{out} ('shadow index') and **b** the in-degrees k_{in} ('overshadow index') at different tropical forest sites (BCI of 50 ha, Sinharaja and Fushan of 25 ha, respectively). In **c** and **d** node degrees at BCI are divided into proportions of shade-tolerant (blue) and light-demanding species (yellow).

a Pioneer trees **b** Climax trees

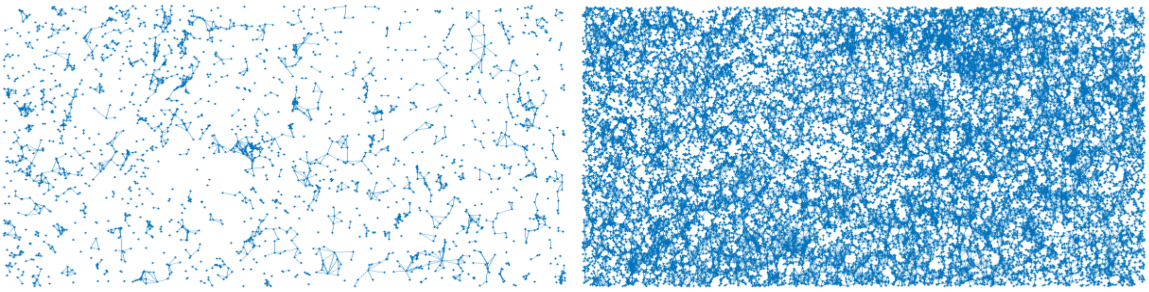

Supplementary Fig. S10 Visualization of a network of pioneer trees (light-demanding trees) and b network of climax trees (shade-tolerant trees) at BCI (50 ha) censused in year 2010. The tree network in **a** includes 2269 trees (nodes) and 511 components (connections of trees isolated from others), while the tree network in **b** consists of 18466 climax trees and 40 components (the largest component contains 18383 trees). The positions of the visualized nodes correspond to the spatial positions of the trees at the forest site.

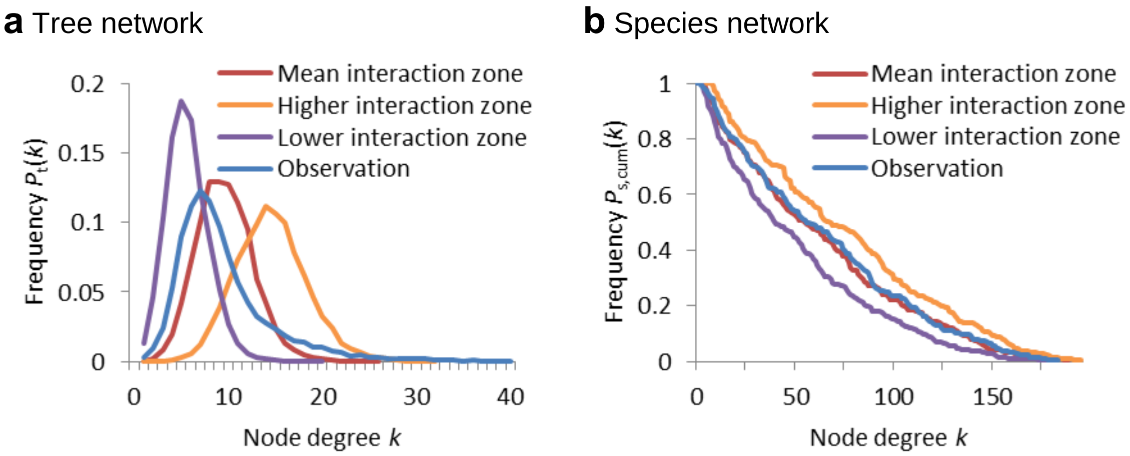

Supplementary Fig. S11 Node degree distributions of **a** the tree networks $P_t(k)$ and **b** the species networks $P_{s,cum}(k)$ (cumulative distribution) for BCI (25 ha, left side) in comparison to networks of ED null communities (Equal interaction Diameter) with tree interaction zones that are equal to the mean interaction zone (red line, interaction diameter $\bar{d}_{int}$ = 20.7 m), lower than the mean interaction zone (purple line, $\bar{d}_{int}$ = 15.9 m) and higher than the mean interaction zone (orange line, $\bar{d}_{int}$ = 25.3 m). In **a** node degrees are cut at k = 40 for graphical aspects.

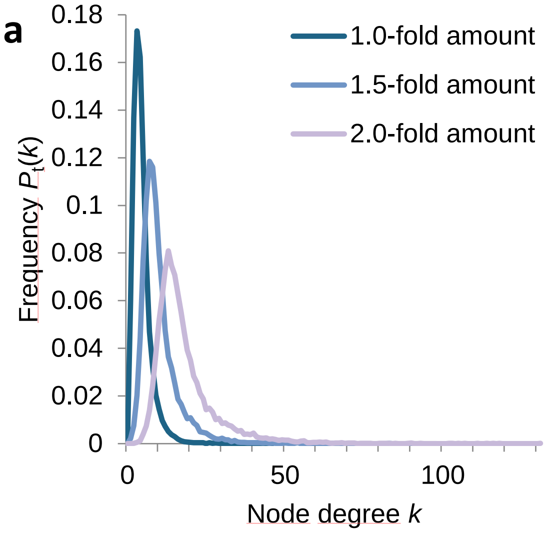

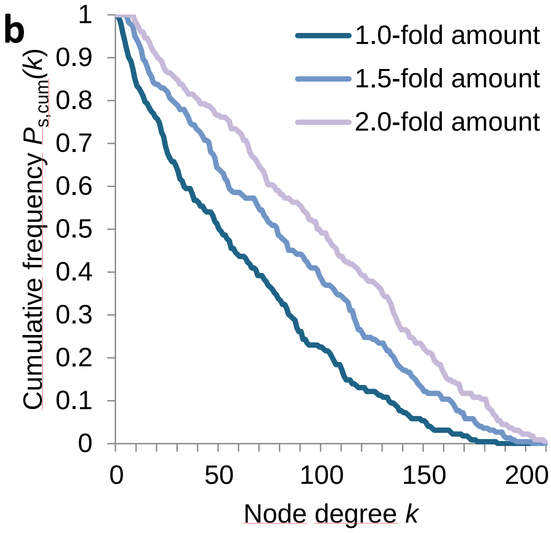

Supplementary Fig. S12 a Node degree distributions $P_t(k)$ of the tree network and **b** cumulative node degree distributions $P_{s,cum}(k)$ of the species network at BCI (50 ha) with different proportionality factors f for deriving the size of interaction zones of trees.

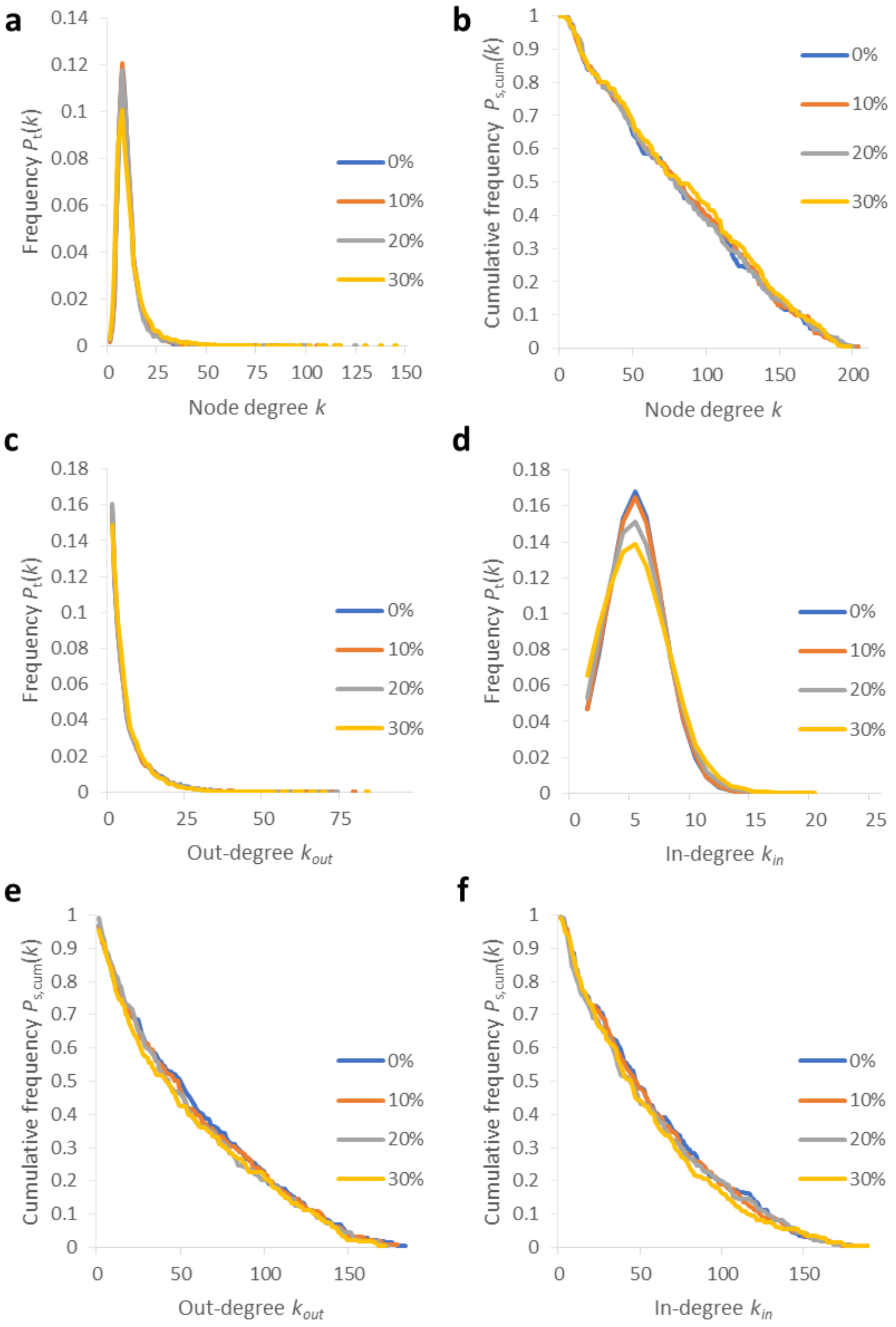

Supplementary Fig. S13 Node degree distributions with noisy interaction zones and tree heights at BCI (50 ha). In **a** and **b** noise was added to parameters of the allometric relationship for the interaction zones per tree species of **a** undirected tree networks and **b** undirected species networks. In **c**-**f** noise was added to parameters of the allometric relationship for tree heights of **c**-**d** directed tree networks (out-degrees and in-degrees) and **e**-**f** directed species networks (out-degrees and in-degrees).

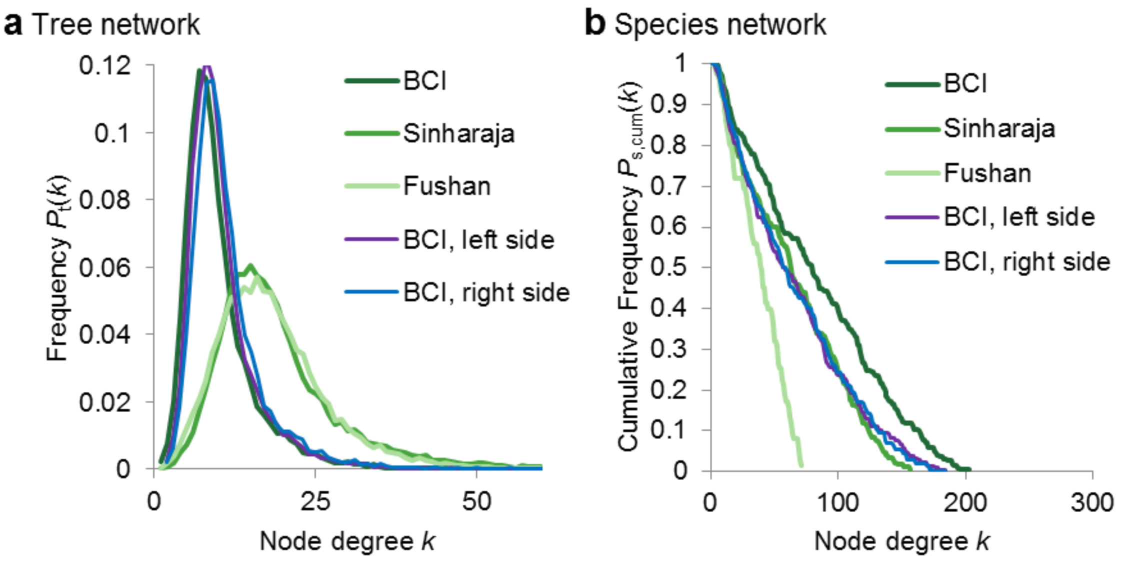

Supplementary Fig. S14 Node degree distributions of **a** the tree networks $P_t(k)$ and **b** the species networks $P_{s,cum}(k)$ (cumulative distribution) for BCI (50 ha), left side of BCI (25 ha), right side of BCI (25 ha), Sinharaja (25 ha) and Fushan (25 ha), respectively. In **a** node degrees are cut at k = 60.

Supplementary Table S1 Summary of undirected tree and species networks for three different forest sites (BCI, Sinharaja, Fushan). Different plot sizes and locations at BCI.

	Forest site	Plot size (ha)	N	E	D	<*k*>	k_{max}	C	L	d
Tree network	BCI (left + right side)	50	20730	104795	0.00049	10.1	98	0.633	31.6	86
	BCI, left side	25	10161	48961	0.00095	9.6	83	0.631	22.6	56
	BCI, right side	25	10567	55419	0.00099	10.5	98	0.637	20.6	58
	Sinharaja	25	17015	163266	0.00113	19.2	136	0.635	21.3	53
	Fushan	25	17647	161285	0.00104	18.3	88	0.630	22.6	58
Species network	BCI (left + right side)	50	222	9201	0.375	82.9	203	0.80	1.6	3
	BCI, left side	25	208	6799	0.316	65.4	184	0.77	1.7	3
	BCI, right side	25	198	6522	0.334	65.9	174	0.78	1.7	3
	Sinharaja	25	177	5727	0.368	64.7	157	0.81	1.6	3
	Fushan	25	75	1404	0.506	37.4	71	0.86	1.5	3

N: number of trees or species (nodes), E: number of connections (edges), D: network density, <*k*>: average node degree, k_{max}: maximal node degree, C: clustering coefficient, L: average path length, d: diameter of the network.

Supplementary Table S2 Results for the species network in BCI (50 ha) assuming different edge thresholds.

Minimum number of interacting trees for an edge	Number of nodes (*N*)	<*k*>	*D*	Local connectivity		Global connectivity	
				C	*C_{ER}	*L*	*L_{ER}
1	222	83	0.374	0.799	0.3757	1.64	1.63
2	212	58	0.274	0.807	0.276	1.76	1.72
4	185	42	0.227	0.826	0.227	1.80	1.77
6	174	33	0.190	0.821	0.190	1.85	1.81
8	159	29	0.182	0.801	0.186	1.87	1.82
10	146	26	0.178	0.808	0.183	1.87	1.83
20	110	19	0.173	0.815	0.169	1.84	1.85
30	94	14	0.149	0.762	0.151	1.90	1.94
40	78	13	0.167	0.796	0.148	1.85	1.92
50	69	11	0.159	0.773	0.170	1.87	1.95

Trees of a species must interact with a minimum number of trees of another species for being considered as interacting. With increasing minimum number the network size *N* and average node degree <*k*> becomes smaller, while the clustering coefficient *C*, average path length *L* and especially small world property remains unchanged.
*C_{ER} and L_{ER}: clustering coefficient and average path length of random graphs following the ER model of the same network size.

Supplementary Table S3 Properties of the analyzed null communities.

Null community	Remains unchanged	Changes
CSR – Complete Spatial Randomness	Species identities<br>Tree size distribution<br>Species abundances	Tree positions are spread evenly distributed on plot
RL – Random Labeling	Tree positions<br>Tree size distribution<br>Species abundances	Species identities are randomly relabeled
ED – Equal interaction Diameter	Tree positions<br>Species identities<br>Species abundances	Tree sizes are set equal to the mean tree size
RGN – Random Geometric Network (CSR + ED)	(Species identities)<br>Species abundances	Tree positions are spread evenly distributed on plot<br>Tree sizes are set equal to the mean tree size

Supplementary Table S4 Comparison between the characteristics of the species networks at three tropical forest sites (size 25 ha) and of analyzed null communities.

		N	E	D	$<k>$	k_{max}	C	L	d	C_{ER}	L_{ER}
BCI (left side)	Observation	208	6799	0.3158	65.4	184	0.772	1.69	3	0.314	1.7
	ED	208	6638	0.3083	63.8	189	0.775	1.70	3	0.307	1.7
	CSR	208	7365	0.3423	70.8	189	0.793	1.66	3	0.343	1.7
	RGN	208	7202	0.3323	69.0	198	0.789	1.67	3	0.334	1.7
	RL	208	7022	0.3262	67.5	194	0.791	1.68	3	0.325	1.7
Sinharaja	Observation	177	5727	0.3677	64.7	157	0.810	1.64	3	0.367	1.6
	ED	177	5939	0.3813	67.1	162	0.807	1.63	3	0.382	1.6
	CSR	177	6550	0.4205	74.0	173	0.843	1.58	3	0.421	1.6
	RGN	177	6717	0.4313	75.9	173	0.841	1.57	3	0.433	1.6
	RL	177	6751	0.4334	76.2	172	0.842	1.57	3	0.432	1.6
Fushan	Observation	75	1404	0.5059	37.4	71	0.856	1.50	3	0.513	1.5
	ED	75	1470	0.5297	39.2	70	0.854	1.47	3	0.533	1.5
	CSR	75	1480	0.5332	39.5	69	0.862	1.47	2	0.534	1.5
	RGN	75	1555	0.5602	41.5	73	0.862	1.44	2	0.560	1.4
	RL	75	1575	0.5677	42.0	73	0.863	1.43	2	0.565	1.4

CSR (complete spatial randomness) and RL (random labeling) are null communities affecting random tree positions (CSR) and random shuffling of existing species identities among trees (RL). ED (Equal Diameters) are null communities with equal interaction diameters (mean over observed interaction diameters) and RGN (Random geometric network) combines CSR and ED (averages of 19 simulations). All networks show the small-world property. N: number of nodes, E: number of edges, D: network density, $<k>$: mean node degree, k_{max}: maximal node degree, C: clustering coefficient, L: average path length, d: diameter of the network, C_{ER} and L_{ER} clustering coefficient and average path length of random graphs following the ER model of the same size.

Supplementary Table S5 Comparison between characteristics of tree networks at three tropical forest sites (size 25 ha) and of related null communities.

		N	E	D	$<k>$	k_{max}	C	L	d
BCI (left side)	Observation	10161	48961	0.0010	9.6	83	0.631	22.6	56
	ED	10163	48962	0.0010	9.6	26	0.566	39.1	102
	CSR	10149	50727	0.0010	10.0	85	0.652	23.2	61
	RGN	10607	54722	0.0010	10.3	24	0.587	38.8	103
Sinharaja	Observation	17015	163266	0.0011	19.2	136	0.635	21.3	53
	ED	17016	163856	0.0011	19.3	39	0.588	33.4	88
	CSR	17034	158505	0.0011	18.6	132	0.642	20.5	54
	RGN	17034	158487	0.0011	18.6	37	0.593	33.3	89
Fushan	Observation	17647	161285	0.0010	18.3	88	0.630	22.6	58
	ED	17646	171714	0.0011	19.5	47	0.592	34.9	98
	CSR	17649	145337	0.0009	16.5	106	0.636	22.9	60
	RGN	17649	144860	0.0009	16.4	35	0.592	36.6	98

ED (Equal Diameters) are null communities with equal interaction diameters (mean over observed interaction diameters) and CSR (complete spatial randomness) are null communities affecting random tree positions. RGN (Random geometric network) combines ED and CSR (averages of 19 simulations). N: number of nodes, E: number of edges, D: network density, $<k>$: mean node degree, k_{max}: maximal node degree, C: clustering coefficient, L: average path length, d: diameter of the network.

Supplementary Table S6 Parameters of the allometric relationships to derive tree height and tree crown diameter (interaction zone) from stem diameter *dbh* (equation (1) and (2)) for each forest site[4,13].

	Tree height allometry		Tree crown diameter allometry	
	h_1	h_2	i_1	i_2
BCI	2.74	0.60	0.37	0.67
Sinharaja	2.78	0.69	0.40	0.66
Fushan	2.74	0.60	0.37	0.67

Supplementary Table S7 Results of network analysis assuming different proportionality factors *f*.

	Proportionality factor *f*	*N*	*E*	*D*	*C*	*L*
Tree network	1.0-fold amount	18285	41648	0.00025	0.567	84.37
	1.5-fold amount	20730	104795	0.00049	0.633	31.61
	2.0-fold amount	20735	188905	0.00088	0.641	19.54
Species network	1.0-fold amount	222	6724	0.274	0.763	1.76
	1.5-fold amount	222	9201	0.375	0.799	1.63
	2.0-fold amount	222	11052	0.451	0.816	1.55

N: number of trees or species (nodes), *E*: number of connections (edges), *D*: network density, *C*: clustering coefficient, *L*: average path length.

Supplementary Table S8 Results of network analysis assuming different noise extents on the interaction zones of tree individuals in BCI (50 ha).

	Noise extent	*N*	*E*	*D*	<*k*>	k_{max}	*C*	*L*	*d*	$\bar{d}_{int}$	$\bar{d}_{shifted}$
Tree network	± 0 %	20730	104795	0.00049	10.1	98	0.633	31.6	86	8.56	8.56
	± 10 %	20724	106175	0.00049	10.2	142	0.642	28.5	76	8.38	8.56
	± 20 %	20720	111002	0.00052	10.7	179	0.666	22.4	62	8.74	8.56
	± 30 %	20719	118562	0.00055	11.4	179	0.696	19.8	54	8.56	8.56
Species network	± 0 %	222	9201	0.375	82.9	203	0.80	1.6	3	8.56	8.56
	± 10 %	222	9254	0.377	83.4	200	0.80	1.6	3	8.38	8.56
	± 20 %	222	9610	0.392	86.6	198	0.80	1.6	3	8.74	8.56
	± 30 %	222	8779	0.358	79.1	209	0.81	1.6	3	8.56	8.56

N: number of nodes, *E*: number of edges, *D*: network density, <*k*>: mean node degree, k_{max}: maximal node degree, *C*: clustering coefficient, *L*: average path length, *d*: diameter of the network, $\bar{d}_{int}$: mean interaction diameter of trees before linear shift [m], $\bar{d}_{shifted}$: mean interaction diameter of trees after linear shift [m].

Supplementary Table S9 Results of network analysis assuming different noise extents on the height of tree individuals in BCI (50 ha).

	Noise extent	N	E	D	$<k>$	$k_{max,in}$	$k_{max,out}$	C_{in}	C_{out}
Tree network	± 0 %	20730	104795	0.00024	5.1	16	98	0.350	0.132
	± 10 %	20730	104795	0.00024	5.1	16	98	0.347	0.136
	± 20 %	20730	104795	0.00024	5.1	18	98	0.340	0.144
	± 30 %	20730	104795	0.00024	5.1	25	98	0.326	0.159
Species network	± 0 %	222	12536	0.256	56.5	189	179	0.704	0.660
	± 10 %	222	12200	0.249	55.0	181	173	0.684	0.656
	± 20 %	222	11824	0.241	53.3	189	172	0.671	0.596
	± 30 %	222	12536	0.256	56.5	189	179	0.704	0.660

N: number of trees or species (nodes), E: number of connections (edges), D: network density, $<k>$: average node degree, $k_{max,in}$/$k_{max,out}$: maximal node degrees of the directed networks, C_{in}/C_{out}: clustering coefficients of the directed networks. Subscripted characters denote network attributes with regard to the in-degrees (‘overshadow indices’) and out-degrees (‘shadow indices’).

Supplementary Table S10 Summary of directed tree and species networks for three different forest sites (BCI, Sinharaja, Fushan). Different plot sizes and locations at BCI.

	Forest site	N	E	D	$<k>$	$k_{max,in}$	$k_{max,out}$	C_{in}	C_{out}
Tree network	BCI (left + right side)	20730	104795	0.00024	5.1	16	98	0.350	0.132
	BCI, left side	10161	48961	0.00047	4.8	16	83	0.346	0.131
	BCI, right side	10567	55419	0.00050	5.2	16	98	0.354	0.133
	Sinharaja	17015	163266	0.00056	9.6	26	136	0.356	0.181
	Fushan	17647	161285	0.00052	9.1	29	88	0.347	0.188
Species network	BCI (left + right side)	222	12776	0.260	57.5	188	184	0.716	0.659
	BCI, left side	208	9153	0.213	44.0	169	155	0.662	0.635
	BCI, right side	198	8662	0.222	43.7	166	156	0.653	0.618
	Sinharaja	177	8710	0.280	49.2	142	147	0.780	0.712
	Fushan	75	2340	0.422	31.2	60	69	0.860	0.745

N: number of trees or species (nodes), E: number of connections (edges), D: network density, $<k>$: average node degree, $k_{max,in}$/$k_{max,out}$: maximal node degrees of the directed networks, C_{in}/C_{out}: clustering coefficients of the directed networks. Subscripted characters denote network attributes with regard to the in-degrees (‘overshadow indices’) and out-degrees (‘shadow indices’).

References

1 Tsai, C.-H. *et al.* Individual species-area relationship of woody plant communities in a heterogeneous subtropical monsoon rainforest. *PLoS ONE* **10**, e0124539, doi:http://dx.doi.org/10.1371/journal.pone.0124539 (2015).

2 Losos, E. C. & Leigh, E. G. *Tropical forest diversity and dynamism.* (University of Chicago Press, Chicago, 2004).

3 Condit, R., Hernandez, A., Perez, R., Lao, S., Angehr, G., Hubbell, S. P. & Foster, R. B. Tropical forest dynamics across a rainfall gradient and the impact of an El Nino dry season. *Journal of Tropical Ecology* **20**, 51-72 (2004).

4 Su, S. H. *et al. Fushan subtropical forest dynamics plot: tree species characteristics and distribution patterns*. (Taiwan Forestry Research Institute, 2007).

5 Bohlman, S. & O'Brien, S. Allometry, adult stature and regeneration requirement of 65 tree species on Barro Colorado Island, Panama. *Journal of Tropical Ecology* **22**, 123-136, doi:http://dx.doi.org/10.1017/S0266467405003019 (2006).

6 Schenk, H. J. & Jackson, R. B. Rooting depths, lateral root spreads and below-ground/above- ground allometries of plants in water-limited ecosystems. *Journal of Ecology* **90**, 480-494 (2002).

7 Barabási, A.-L. *Network science*. (Cambridge University Press, 2016).

8 Virkar, Y. & Clauset, A. Power-law distributions in binned empirical data. **8**, 89-119, doi:10.1214/13-AOAS710 (2014).

9 Muller-Landau, H. C. *et al.* Comparing tropical forest tree size distributions with the predictions of metabolic ecology and equilibrium models. *Ecology Letters* **9**, 589-602 (2006).

10 Enquist, B. J. & Niklas, K. J. Invariant scaling relations across tree-dominated communities. *Nature* **410**, 655-660 (2001).

11 Provero, P. (arXiv:cond-mat/0207345v2 [cond-mat.stat-mech], 2002).

12 Herrmann, C., Barthélemy, M. & Provero, P. Connectivity distribution of spatial networks. *Physical Review E* **68**, 026128, doi:10.1103/PhysRevE.68.026128 (2003).

13 Kohyama, T., Suzuki, E., Partomihardjo, T., Yamada, T. & Kubo, T. Tree species differentiation in growth, recruitment and allometry in relation to maximum height in a Bornean mixed dipterocarp forest. *Journal of Ecology* **91**, 797-806 (2003).
